# Spezialisierte ambulante Palliativversorgung bei urologischen Tumoren

**DOI:** 10.1007/s00120-025-02573-x

**Published:** 2025-04-04

**Authors:** Stephan Degener, Nici Markus Dreger, Friedrich-Carl von Rundstedt, Marie-Therese Schmitz, Ulrich Grabenhorst, Oliver Schmalz, Klaus Weckbecker, Johannes Just

**Affiliations:** 1 Klinik für Urologie und Kinderurologie, Helios Universitätsklinikum Wuppertal, Universität Witten/Herdecke, Heusnerstraße 40, 42283 Wuppertal, Deutschland; 2 Institut für Medizinische Biometrie, Informatik und Epidemiologie (IMBIE), Medizinische Fakultät, Universität Bonn, Bonn, Deutschland; 3 Verbund der SAPV-Teams in Nordrhein e. V., Solingen, Deutschland; 4 Klinik für Hämatologie, Onkologie, klinische Infektiologie und Palliativmedizinx, Helios Universitätsklinikum Wuppertal, Universität Witten/Herdecke, Wuppertal, Deutschland; 5 Institut für Allgemeinmedizin und ambulante Gesundheitsversorgung (IAMAG), Universität Witten/Herdecke, Witten, Deutschland

**Keywords:** Krebs, Palliativmedizin, Symptommanagement, SAPV, Pflege am Lebensende, Cancer, Palliative care, Symptom management, SAPV, End-of-life care

## Abstract

**Hintergrund:**

Urologische Tumoren umfassen gut 20 % der Krebsdiagnosen in Deutschland. Über die ambulante Palliativversorgung von Patienten mit urologischen Tumoren im häuslichen Umfeld gibt es bisher keine Untersuchungen großer Kohorten, obwohl eine würdevolle letzte Lebensphase im häuslichen Umfeld der Wunsch der meisten Krebspatienten ist.

**Methode:**

Daten von 5125 Patienten, die zwischen 2017 und 2021 in der spezialisierten ambulanten Palliativversorgung (SAPV) mit einer uroonkologischen Grunderkrankung behandelt wurden, gingen in die Analyse ein.

**Ergebnis:**

Die Analyse ergab, dass 91,5 % der Patienten im häuslichen Umfeld verstarben und die Symptomlast während der Behandlung stabil blieb oder leicht abnahm. Die Überlebenszeit in der SAPV betrug im Median 24 Tage. Faktoren wie Alter, Leistungsfähigkeit, Appetitlosigkeit, Schwäche und Schmerzen zu Beginn der Behandlung beeinflussten die Überlebenszeit der Patienten. Angehörige zeigten sich mit der Versorgung größtenteils sehr zufrieden.

**Schlussfolgerung:**

Die Studie liefert wichtige Erkenntnisse zur palliativen Versorgung von Patienten mit urologischen Tumoren und unterstreicht die Bedeutung der SAPV für eine würdevolle Betreuung am Lebensende.

**Graphic abstract:**

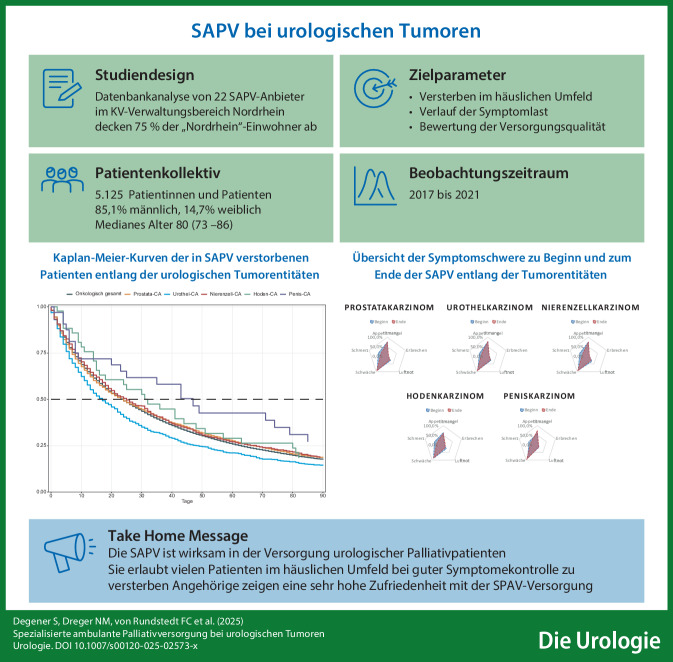

## Abstract (en)

**Background:**

Urologic tumors comprise about 20% of cancer diagnoses in Germany. To date, there are no studies with large datasets on the palliative situation of patients with urological tumors in the home environment, although dignified end-of-life care in the home environment is the wish of most cancer patients.

**Methods:**

Data from 5125 patients who received specialized outpatient palliative care (SAPV) with an underlying urological cancer between 2017 and 2021 were included in the analysis.

**Result:**

The analysis showed that 91.5% of patients died at home and the symptom burden remained stable or decreased slightly during treatment. The median survival time of patients receiving SAPV was 24 days. Factors such as age, performance status, loss of appetite, weakness, and pain at the start of treatment influenced the survival time of the patients. Relatives were largely very satisfied with the care provided.

**Conclusion:**

The study provides important insights into the palliative care of patients with urological tumors and underlines the importance of SAPV for dignified end-of-life care.

## Hintergrund und Fragestellung

Bereits heute ist mehr als jedes fünfte (20,8 %) neudiagnostizierte Karzinom in Deutschland urologischen Ursprungs. Das entspricht etwas über 100.000 Neuerkrankten pro Jahr [1]. Bei den Krebssterbefällen entfallen 11,4 % auf urologische Malignome [1].

Prognosen des Robert-Koch-Instituts (RKI) und der Gesellschaft der Epidemiologischen Krebsregister in Deutschland (GEKID) gehen jedoch von einer weiter steigenden Zahl an Krebspatienten aus. Uroonkologisch sind davon das Prostata- und das Harnblasenkarzinom besonders betroffen, hier wird ein Anstieg um ca. 25 % bis zum Jahr 2050 erwartet [2].

Diese Daten verdeutlichen die Bedeutung der Palliativmedizin: Die Deutsche Gesellschaft für Palliativmedizin (DGP) schätzt, dass bis zu 90 % aller Verstorbenen pro Jahr einen palliativen Versorgungsbedarf und ca. 10 % wegen einer besonders aufwändigen Versorgungssituation einen Bedarf für die *spezialisierte ambulante Palliativversorgung* (SAPV) haben [3].

Die SAPV stellt einen wichtigen Bestandteil im palliativmedizinischen Versorgungskonzept in Deutschland dar. Ihr Ziel ist es, Patienten auch dann weiter im häuslichen Wohnumfeld zu versorgen, wenn die hausärztliche oder die allgemeine ambulante Palliativversorgung (AAPV) nicht mehr als ausreichend bewertet werden. Sie ist auf die Behandlung von Patienten mit besonderem Versorgungsaufwand und/oder besonders schwerwiegenden Symptome ausgelegt [4, 5].

Analysen größerer SAPV-Kohorten auf Länder- wie auf Bundesebene zeigen, dass insbesondere beim ideellen Hauptziel der SAPV, einem Versterben im gewünschten Wohnumfeld unter bestmöglicher Symptomkontrolle, viele Patienten vom Einsatz der SAPV profitieren können [6].

Bisher liegen keine Analysen zur Arbeit der SAPV auf dem Gebiet der urologischen Tumoren vor. Dabei können diese Analysen eine wertvolle Hilfestellung sein, häufig vorkommende Beschwerden in der Palliativsituation frühzeitig in der ambulanten uroonkologischen Betreuung zu erkennen und in das Behandlungskonzept zu integrieren.

## Studiendesign und Untersuchungsmethoden

### Datenquelle

Es erfolgt die pseudonymisierte Datenextraktion von 22 SAPV-Anbietern, die im Verwaltungsbereich der Kassenärztlichen Vereinigung (KV) Nordrhein in den Jahren 2017 und 2021 tätig waren. Über die definierten geographischen Zuständigkeitsgebiete dieser 22 SAPV-Anbieter werden gut 7,2 Mio. (75 %) der rund 9,5 Mio. Einwohner im KV-Bereich Nordrhein abgedeckt [7].

Bei den erfassten Daten handelt es sich um routinemäßig erhobene Daten zur Falldokumentation. Die Datenextraktion wurde durch den Verbund der SAPV-Teams Nordrhein e. V. (VSTN) koordiniert, in dem alle beteiligten Datengeber Mitglied sind.

Die für die Studie erfassten Patientendaten wurden aus den elektronischen Akten in anonymisierter Form extrahiert. Die anschließende Zusammenführung der Daten sowie das Datenqualitätsmanagement erfolgte über StatConsult (StatConsult Gesellschaft für klinische und Versorgungsforschung mbH, Magdeburg, Deutschland), einem Softwareentwickler und Vertragsinstitut für Aufgaben in der klinischen Forschung, Entwicklung und Versorgungsforschung.

Bei dem Forschungsvorhaben wurden die Vorgaben der aktuellen Fassung der Deklaration von Helsinki stets beachtet. Zu Beginn des Forschungsvorhabens wurde das Projekt der Ethikkommission der Ärztekammer Nordrhein vorgelegt. Hier wurden keine ethischen Bedenken oder Beanstandungen ausgesprochen (Antrag Nr. 48/2023).

### Ein- und Ausschlusskriterien

In die Auswertung eingeschlossen wurden alle Patienten, die zwischen 2017 und 2021 in Behandlung eines der 22 SAPV-Teams waren. Dabei wurden auch Fälle berücksichtig, die 2017 endeten (aber vorher begonnen hatten) oder Fälle die noch 2021 eingeschlossen wurden (aber erst später endeten).

Die Information zum Sterbeort der Patienten setzt sich aus Angaben zum Entlassungsgrund aus dem letzten SAPV-Versorgungszeitraum („verstorben“) und/oder dem Vorliegen einer Dokumentation zum Sterbeort nach Entlassung aus der SAPV zusammen.

In der Routine erfolgen SAPV-Behandlungen kontinuierlich (ohne Pause zwischen Versorgungsstart und Sterbezeitpunkt). Aus unterschiedlichen Gründen kann es jedoch zu Pausen oder zur Beendigung der SAPV kommen. Gründe dafür sind in aller Regel Krankenhausaufenthalte, Verlegungen in ein Hospiz oder die Verbesserung der Symptomlast durch die intensivierte Betreuung.

Etwa ein Fünftel der Patienten in unserem Datensatz wies eine oder mehrere Pausen auf. Da es sich aber aus klinischer Sicht meist um einen zusammenhängenden klinischen Verlauf handelt, dessen kassenrechtlicher Status lediglich aus bürokratischen Notwendigkeiten heraus kurzzeitig pausiert wird, wurde definiert, Fälle zusammenzufassen, bei denen die Behandlungspausen < 13 Tagen dauerten. Hintergrund dieser Entscheidung ist, dass meist Krankenhausaufenthalte für spezialisierte Verfahren wie palliative Chemotherapie, Radiotherapie oder Transfusionen für die Pausen verantwortlich waren.

Auch wenn der Cut-off von 13 Tagen aufgrund von Expertenmeinungen gewählt wurde, war die Entscheidung zudem auch statistisch gestützt, da die Pausen bei circa 90 % der Fälle kleiner als 13 Tage waren.

### Quantitative Variablen und statistische Analyse

Aus der Gesamtkohorte (48.882 Patienten zwischen 2017 und 2021) wurden alle Fälle nach ICD 10 selektiert, die wegen einer uroonkologischen Diagnose als Haupt- oder Aufnahmediagnose in der SAPV behandelt wurden. Diese waren im Einzelnen: C60 (Peniskarzinom), C62 (Hodenkarzinom), C61 und D40.0 (Prostatakarzinom), C64 und D41.0 (Nierenzellkarzinom), C65 und D41.1 (Nierenbeckenkarzinom), C66 und D41.2 (Harnleiterkarzinom) sowie C67, D41.4, D09.0 und D09.1 (Harnblasenkarzinom).

Ziel der vorliegenden Analyse war eine deskriptive Analyse der Versorgung von Patienten mit urologischen Tumoren in der SAPV unter Zuhilfenahme relevanter klinischer Endpunkte:

- „Patientenwunsch“: Versterben im häuslichen Wohnumfeld,
- „Symptomkotrolle“: Verlauf der Symptomlast,
- „Zufriedenheit“: Einschätzung der Versorgungsqualität durch Dritte.

Analysen zu Veränderungen in der Symptomlast zu Beginn und zum Ende der Betrachtung erfolgten mittels McNemar-Test. Für die Überlebenszeitanalysen (von Eintritt in die SAPV bis zum Tod) wurden Kaplan-Meier-Schätzungen vorgenommen, sowohl in der Analyse der Gesamtgruppe als auch der Subgruppen entlang der Tumorentitäten.

Der Ort des Versterbens wurde in ambulant bzw. „häusliches Umfeld“ versus stationär, bzw. „Aufenthalt Krankenhaus“ unterschieden. Als ambulant bzw. „häusliches Wohnumfeld“ wurden neben der eigenen Wohnung auch Senioren- und Pflegeheime, Kurzzeitpflege, Hospiz und sonstige Aufenthaltsorte definiert, da es sich jeweils um selbstbestimmte Wohnorte handelt, an denen die SAPV als ärztliche Teilleistung erbracht werden kann. In Abgrenzung dazu wurden als stationär bzw. „Aufenthalt Krankenhaus“ alle Aufenthalte in Akutkrankenhäusern und/oder Palliativstation gewertet.

Für die Regressionsanalyse wurde der Sterbeort dichotomisiert als „häusliches Wohnumfeld“ versus „Aufenthalt Krankenhaus“ betrachtet. Der Einschluss verschiedener Variablen auf die Überlebenszeit in der SAPV wurde mittels Cox-Regression analysiert und adjustierte Hazard Ratios mit 95-%-Konfidenzintervallen (KI) berechnet. Die graphische Darstellung der Assoziationen erfolgte mittels Forest-Plot.

Um eine zusätzliche Einschätzung der Versorgungsqualität zu gewährleisten wurde die Variable „Behandlungszufriedenheit“ mit eingeschlossen. Hierzu wurde nach dem Versterben der Patientin bei einem Teil der Teams die Zufriedenheit der Angehörigen bzw. der betreuenden Mitarbeiter befragt (5-Punkte-Skala: sehr gut bis sehr schlecht). Zur Vereinfachung der Auswertung wurden diese Werte gepoolt.

## Ergebnisse

Von den 48.882 Patienten, die in dem 5‑Jahres-Zeitraum (2017–2021) in der SAPV behandelt wurden, wiesen 5125 Patienten eine urologische Krebsdiagnose als Haupt- oder Aufnahmediagnose auf. Wie zu erwarten, wiesen über die Hälfte der Betroffenen ein Prostatakarzinom als Krebsdiagnose auf (61,1 %), gefolgt vom Urothelkarzinom (25,7 %) und dem Nierenzellkarzinom (17,1 %). Die genaue Verteilung findet sich in Tab. 1.

**Tab. 1:** Absolute und relative Häufigkeiten der uroonkologischen Aufnahme- und Hauptdiagnosen sowie das mediane Alter mit Angabe der Interquartilabstände (IQR). Hinweis: Es gibt mehrere Personen, die mehr als eine der Diagnosen aufweisen, daher summieren sich die Zahlen auf mehr als 100 %

Tumorentität	Anzahl (*n*, %)		Alter in Jahren (Median, IQR)
Prostatakarzinom	3129	61,1	81 (75–86)
Urothelkarzinom	1315	25,7	81 (74–87)
*Nierenbeckenkarzinom*	*69*	*1,3*	
*Harnleiterkarzinom*	*48*	*0,9*	
*Harnblasenkarzinom*	*1198*	*23,4*	
Nierenzellkarzinom	877	17,1	78 (69–84)
Hodenkarzinom	57	1,1	60 (49–73)
Peniskarzinom	45	0,9	79 (72–85)

Passend zum zahlenmäßig führenden Prostatakarzinom, waren 85,1 % (*n* = 4363) der Patienten in der Studie männlich und nur 14,7 % (*n* = 751) weiblich. Das mediane Alter lag bei 80 Jahren (Q1-Q3: 73–86), wobei der jüngste Patient 19 und der älteste 107 Jahre alt war.

Während bei fast allen Patienten in der End-of-life-Situation auf Angehörige zurückgegriffen werden konnte (95,1 %, *n* = 4874), lag nur bei knapp 60 % eine Vollmacht vor und nur gut jede/r zweite Patienten (51,7 %, *n* = 2651) hatte eine Patientenverfügung.

Der mediane Charlson Comorbidity Index (CCI) lag bei 8 (4–9), der altersangepasste CCI [8] bei Median 11 (8–12). Bei gut der Hälfte der Patienten (51,7 %, *n* = 2649) lag eine weitere Tumorerkrankung als Nebendiagnose vor (Tab. 2 gibt einen Überblick über Charakteristika der Gesamtkohorte).

**Tab. 2:** Absolute und relative Häufigkeiten der wichtigsten Parameter der Gesamtkohorte

Charakteristika der Gesamtkohorte		Gesamt (*n* = 5125)	
Geschlecht (*n*, %)	Männlich	4363	85,1
	Weiblich	751	14,7
	Nicht bekannt	11	0,2
Alter [Jahre] (bei Beginn aktive SAPV)	Median (Q1–Q3)	80 (73–86)	
	Min; Max	19	107
Angehörige/Vertrauensperson (*n*, %)	Ja	4874	95,1
	Nein	251	4,9
Vollmacht (*n*, %)	Ja	2976	58,1
	Nein	2149	41,9
Patientenverfügung (*n*, %)	Ja	2651	51,7
	Nein	2474	48,3
Charlson Comorbidity Index (CCI)	Median, Q1–Q3	8	4–9
	Min; Max	0	16
Altersadaptierter Charlson Comorbidity Index (ageCCI)	Median, Q1–Q3	11	8–12
	Min, Max	2	19
Weitere Tumor-Nebendiagnose (*n*, %)	Nein	2476	48,3
	Ja	2649	51,7

### Symptomlast

Hinsichtlich der Funktionalität zeigten sich passend zur Kohorte deutliche Einschränkungen. So wiesen 79,6 % der Patienten zu Beginn der SAPV einem Karnofsky-Index von ≤ 40 % auf, was dauerhaft qualifizierte Hilfe erforderlich macht. Bei 56 % zeigte sich ein Karnofsky von ≤ 30 %, was einer Bettlägerigkeit entspricht. Durch die SPAV wiesen bei der letzten Erhebung signifikant weniger Patienten einen Karnofsky ≤ 30 % auf (50 %, *p* < 0,001).

Bei der Betrachtung der Symptomlast kam es im Behandlungszeitraum zu einer leichten Zunahme von mittelstarker bis starker Schwäche (90,9 % vs. 94,4 %; *p* < 0,001).

**Abb. 1:**
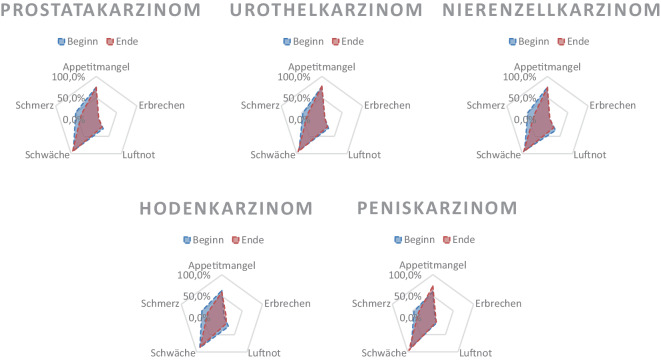
Übersicht der (kombiniert mittleren und schweren) Symptomschwere zu Beginn und zum Ende der SAPV entlang der unterschiedlichen Tumorentitäten aufschlüsselt (Angaben in Prozent)

Gut drei Viertel der Patienten klagten zu Beginn über mittelstarken bis starken Appetitmangel (74,8 %), was sich bis Ende der SAPV auch nicht wesentlich änderte (75,1 %, *p* = 0,776). Deutlich geringer war die Belastung durch Erbrechen, jedoch auch ohne signifikante Veränderung (6,0 % versus 6,3 %; *p* = 0,311). Deutliche Veränderungen zeigten sich bei schwerwiegenden Symptomen Luftnot und Schmerzen: Klagten zu Beginn 28 % über mittlere/starke Luftnot, waren es am Ende der SPAV nur noch 22,9 % (*p*  < 0,001). Mittlere/starke Schmerzen konnten durch die SAPV von 49,1 % zu Beginn auf 35,6 % am Ende reduziert werden (*p*  < 0,001). Tab. 3 gibt einen Überblick über die Symptomschwere zu Beginn und zum Ende des SAPV, während Abb. 1 die Symptomlast zu Beginn und zum Ende der SAPV entlang der unterschiedlichen Tumorentitäten vergleichend darstellt.

**Tab. 3:** Absolute und relative Häufigkeiten des Performance Status nach Karnofsky sowie relevanter Symptome der Gesamtkohorte. Analysen zu Veränderungen in der Symptomlast (kombiniert mittel und stark) erfolgten mittels McNemar-Test

Übersicht Symptomlast				Gesamt (*n* = 5125)	
Karnofsky	(Beginn) (*n*, %)		(Ende) (*n*, %)		*p*-Wert
100	2	0,0	2	0,0	–
90	3	0,1	3	0,1	
80	16	0,3	11	0,2	
70	59	1,2	54	1,1	
60	267	5,2	214	4,2	
50	694	13,5	645	12,6	
40	1207	23,6	1633	31,9	
30	1258	24,5	1480	28,9	< 0,001
20	1099	21,4	727	14,2	
10	520	10,1	356	6,9	
Schwäche	(Beginn) (*n*, %)		(Ende) (*n*, %)		*p*-Wert
Kein	31	0,6	29	0,6	–
Leicht	433	8,4	257	5,0	
Mittel	1931	37,7	1033	20,2	< 0,001
Stark	2730	53,3	3806	74,3	
Appetitmangel	(Beginn) (*n*, %)		(Ende) (*n*, %)		*p*-Wert
Kein	433	8,4	362	7,1	–
Leicht	856	16,7	916	17,9	
Mittel	1561	30,5	1025	20,0	0,776
Stark	2275	44,4	2822	55,1	
Erbrechen	(Beginn) (*n*, %)		(Ende) (*n*, %)		*p*-Wert
Kein	3507	68,4	3800	74,1	–
Leicht	1312	25,6	1001	19,5	
Mittel	222	4,3	234	4,6	0,311
Stark	84	1,6	90	1,8	
Luftnot	(Beginn) (*n*, %)		(Ende) (*n*, %)		*p*-Wert
Kein	1517	29,6	1563	30,5	–
Leicht	2174	42,4	2389	46,6	
Mittel	1069	20,9	850	16,6	< 0,001
Stark	365	7,1	323	6,3	
Schmerzen	(Beginn) (*n*, %)		(Ende) (*n*, %)		*p*-Wert
Kein	794	15,5	1102	21,5	–
Leicht	1816	35,4	2200	42,9	
Mittel	1818	35,5	1255	24,5	< 0,001
Stark	697	13,6	568	11,1

Beide Betrachtungen sollten vor dem Hintergrund eines für den Patienten minimal bedeutsamen Unterschieds („minimal clinically important difference“, MCID) bewertet werden. Während sich z. B. für das Symptom „Schmerz“ sowohl in Gesamtbetrachtung als auch entlang der Tumorentitäten ein stabiler Rückgang von bis > 10 % zeigt (was als bedeutsamer Unterschied gewertet werden darf), sind die Rückgänge bei „Luftnot“ zwar auch über die Tumorentitäten konstant jedoch insgesamt eher gering ausgeprägt und bei anderen Symptomen (Schwäche, Appetitmangel) zeigen sich eher Verschiebungen zwischen den Ausprägungsstärken. Somit zeigt sich insgesamt eine stabile bis rückläufige Symptomlast (für mittlere und starke Symptomschwere), obwohl der natürliche Verlauf terminaler Erkrankungen ein umgekehrtes Verhältnis vermuten lassen würde – was auch als bedeutsamer Unterschied gelten kann.

### Überleben

Die überwiegende Anzahl der Patienten (*n* = 4854, 94,7 %) wohnte bei Aufnahme in die SAPV im definierten häuslichen Wohnumfeld. Von den verstorbenen Patienten sind 91,4 % (*n* = 3664) ebenfalls im häuslichen Umfeld verstorben und nur 8,6 % (*n* = 344) in stationärer Betreuung. Am Ende der Auswertung waren gut 5 von 6 Patienten in der SPAV verstorben.

Über die gesamte Kohorte verbrachten Patienten im Median 18 Tage in SAPV (Q1–Q3 6–50), wobei die Spannweite von keinem ganzen Tag bis hin zu fast 2,5 Jahren (840 Tage) reichte.

**Abb. 2:**
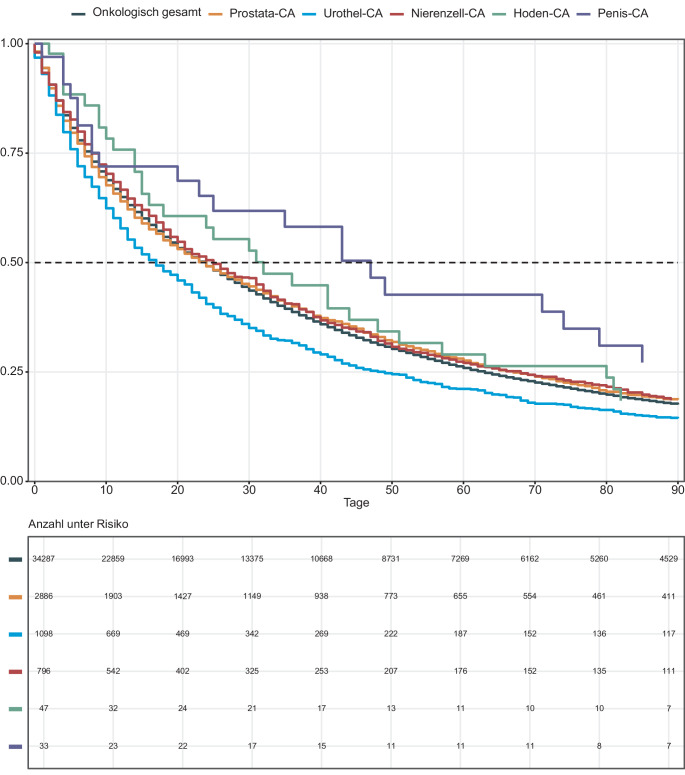
Kaplan-Meier-Kurven (bis 90 Tage) aller in SAPV verstorbenen Patienten entlang der urologischen Tumorentitäten sowie der Gesamtheit der urologischen Tumoren

Betrachtet man das mediane Überleben der an urologischen Tumoren verstorbenen Patienten, bestimmen die häufigsten Entitäten (Prostata‑, Urothel- und Nierenzellkarzinom) das mediane Gesamtüberleben von 24 Tagen. Patienten mit Hoden- und Peniskarzinom leben teilweise doppelt so lang in SAPV (Überlebensdaten s. Tab. 4 und Abb. 2).

**Tab. 4:** Mediane Überlebenszeit mit 95 %-Konfidenzintervall (KI) in SAPV verstorbenen Patienten entlang der Tumorentitäten sowie der Gesamtheit der urologischen Tumoren

Überlebensdaten der Gesamtkohorte			
Tage (in aktiver SAPV)	Median (Q1–Q3)	18 (6–50)	
	Min, Max	0	840
Verstorben (*n*, %)	Nein	785	15,3
	Ja	4340	84,7
Aufenthaltsort bei SAPV-Aufnahme (*n*, %)	Zu Hause	3723	76,1
	Senioren- und Pflegeheim	802	16,4
	Stationäres Hospiz	252	5,1
	Kurzzeitpflege	45	0,9
	Wohngemeinschaften (Beatmung/Demenz/Betreutes Wohnen)	17	0,3
	Sonstige Aufenthaltsorte	15	0,3
	Krankenhaus	36	0,7
	Palliativstation	5	0,1
	Fehlend	230	4,5
Sterbeort (*n*, %)	Zu Hause	2512	62,7
	Senioren und Pflegeheim	684	17,1
	Stationäres Hospiz	434	10,8
	Kurzzeitpflege	31	0,8
	Sonstiger Aufenthaltsort	3	0,1
	Krankenhaus	223	5,6
	Palliativstation	121	3,0
	Personen, die nicht verstorben sind (*n* = 785) und fehlende Angaben	1117	–
Zufriedenheit bei Entlassung (*n*, %)	Sehr gut	2398	76,8
	Gut	650	20,8
	Mittel	61	2,0
	Schlecht	10	0,3
	Sehr schlecht	5	0,2
	Fehlend	2001	39,0
Medianes Überleben Onkologisch gesamt	24 Tage mit 95 %-KI [23–24]		
Medianes Überleben Peniskarzinom	47 Tage mit 95 %-KI [20–79]		
Medianes Überleben Hodenkarzinom	32 mit 95 %-KI [15–48]		
Medianes Überleben Prostatakarzinom	24 mit 95 %-KI [22–26]		
Medianes Überleben Nierenzellkarzinom	25 mit 95 %-KI [21–30]		
Medianes Überleben Urothelkarzinom (Nierenbecken/Harnleiter/Harnblase):	17 mit 95 %-KI [15–20]

### Einflussfaktoren auf die Überlebenszeit

**Abb. 3:**
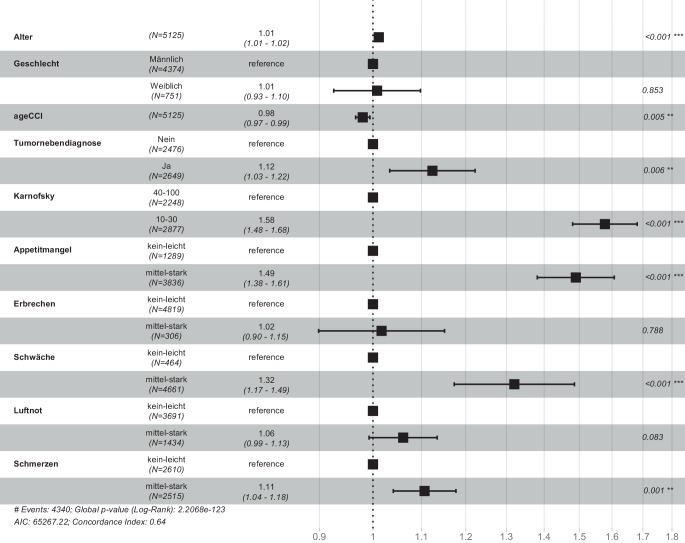
Forest-Plot zu den Ergebnissen des multiplen Modells (Cox-Regression mit allen urologischen Tumoren mit adjustieren Hazard Ratios und 95%-KI)

Abschließend erfolgte eine Cox-Regressionsanalyse zur Frage des Einflusses von relevanten Merkmalen und Symptomen auf die Überlebenszeit in der SAPV. Dabei zeigte sich für die Gesamtheit der urologischen Tumoren ein unabhängiger negativer Einfluss durch das Alter, jedoch nicht durch das Geschlecht der Patienten. Auch war ein Karnofsky zwischen 10–30 mit einem deutlich kürzeren Überleben verbunden. Zudem zeigten mittelstarke und starke Appetitlosigkeit, Schwäche und Schmerzen einen negativen Einfluss. Ohne negativen prognostischen Wert waren mittelstarkes und starkes Erbrechen und Luftnot. Abb. 3 zeigt die Ergebnisse der Cox-Regression.

## Diskussion

Unsere Untersuchung beschreibt erstmals eine große Kohorte von uroonkologischen Patienten in der SAPV. Dabei wird deutlich, dass durch SAPV vielen Patienten ein würdevolles Versterben im gewählten Wohnumfeld bei bestmöglicher Symptomkontrolle ermöglicht werden kann. So verstirbt weniger als jeder 10. Patient in einer stationären Einrichtung. Und obwohl bei terminaler Erkrankung eine Symptomprogression zu erwarten ist, konnten natürliche Symptomverläufe häufig aufgehalten oder verbessert werden. Dazu passend waren zum Ende der Auswertung „nur“ 80 % der Patienten in der SPAV verstorben. Das entspricht auch den Werten anderer Erkrankungen, bei denen knapp 20 % lebend aus der SAPV entlassen werden. Zumeist bei Stabilisierung der Symptome in die Regelversorgung oder wegen Verlegung an einen Ort wo die SAPV nicht betreut.

Diese Erfolge in der Symptomkontrolle spiegeln sich in einer extrem positiven Bewertung der SPAV durch die Hinterbliebenen wider: Mehr als drei Viertel der Mitarbeiter bzw. der Angehörigen bewerteten ihre Zufriedenheit mit der SPAV als sehr gut (76,8 %) und weitere 20,8 % als gut.

Wie auch schon andere Daten zur SAPV in Deutschland [6] aber auch zur ambulanten Palliativmedizin bei urologischen Tumoren in den USA [9] zeigt unsere Analyse einen signifikant höheren Anteil von Patienten, die im häuslichen Umfeld sterben können (91,5 % der verstorbenen Patienten, *n* = 3664) als im Bundesschnitt. Im Jahre 2017 starben lediglich 27 % im häuslichen Wohnumfeld, wenngleich dieser Anteil seit 2001 kontinuierlich zugenommen hat (2001: 21 %; 2011: 23 %; [10, 11]). Insgesamt entspricht die in dieser Studie untersuchte Kohorte hinsichtlich des Alters und des Sterbeorts anderen Vergleichskohorten aus der SAPV [6, 12, 13].

Im Hinblick auf das andere wichtige Ziel, die Linderung von Symptomen, zeigen unsere Daten ebenfalls positive Effekte der SAPV. Schmerzen, Luftnot, Übelkeit, Erbrechen, Schwäche gehören neben Verwirrtheit und Depressionen zu den häufigsten Symptomen in der Palliativmedizin onkologischer Erkrankungen [14, 15].

Durch die SAPV können Symptome wie Schmerzen und Atemnot medikamentös gut behandelt werden und zeigen sich im Verlauf der Betrachtung weniger stark ausgeprägt als zu Beginn. Dies spricht für eine erfolgreiche Pharmakotherapie in der SAPV. Aber auch allgemeine Symptome wie Appetitlosigkeit, Schwäche und Funktionalität konnten (wenn auch auf niedrigem Niveau) trotz progredienter Erkrankung durch die SAPV verbessert werden. Dies wird passt zu internationalen Erfahrungen, die eine Verbesserung der Symptomlast (und der Lebensqualität) bei frühzeitiger Integration der insbesondere ambulanten Palliativmedizin auch bei urologischen Tumoren zeigen [16].

Urologen haben als primäre uroonkologische Behandler eine Schlüsselrolle in der Therapie der urologischen Tumorerkrankungen in allen Stadien der Erkrankung. Das umfasst auch die palliativmedizinische Betreuung der Patienten, wenn die Therapie primär oder im Verlauf nicht mehr in kurativer Intention erfolgen kann. Da viele medikamentöse Tumortherapien erst im palliativen Stadium ihre Indikation haben, spielt die parallele palliativmedizinische Betreuung in der Uroonkologie eine wesentliche Rolle, auch wenn die einschneidenden Symptome zumeist erst im späteren Verlauf der Erkrankung auftreten. Vor diesem Hintergrund scheint eine entsprechende Zusatzqualifikation für Urologen sinnvoll [17, 18] und auch eine direkte Einbindung von Urologen in die SAPV kann für Patienten von großem Vorteil sein [19].

Die Stärke dieser Studie ergibt sich aus der Größe des Datensatzes mit der dadurch möglichen differenzierten Betrachtung der Symptomlast von Menschen mit urologischen Tumoren in der SAPV in Deutschland. Die multizentrische Erhebung über 22 SAPV-Dienstleister in der gesamten Region Nordrhein (aus eher ländlich geprägten Regionen bis zu dicht besiedelten Metropolregionen) kann entsprechend als repräsentativ bewertet werden.

Zudem hat Nordrhein für alle SAPV-Leistungserbringer identische Vorgaben hinsichtlich Struktur- und Prozessqualität, Abrechnung etc., sodass eine Verzerrung durch in der Analyse fehlende Anbieter vernachlässigbar scheint.

Dennoch muss als Limitierung klar benannt werden, dass die SAPV regional weiterhin variiert. Somit muss sowohl die ausschließliche Betrachtung des KV-Bereichs Nordrhein als auch die geringe Inanspruchnahme der SAPV (in Nordrhein bei gut 9 % aller Verstorbenen, und damit etwas niedriger als im Bundesschnitt [3]) bei der Interpretation trotz der Größe der Kohorte berücksichtig werden. Zudem kann aus den vorliegenden Daten nicht herausgearbeitet werden, ob bei allen Patienten, die aufgrund der komplexen Symptomkonstellation und oder des besonderen Versorgungsbedarf ihrer Erkrankung einer SAPV bedurften eine Verordnung von SAPV erfolgte. Aus urologischer Sicht wären zudem Daten zu spezifischen urologischen Symptomen palliativer Tumoren (Stauungsnieren, Hämaturie, Katheter etc.) wünschenswert.

## Fazit für die Praxis

- Die spezialisierte ambulante Palliativversorgung (SAPV) ist wirksam in der Versorgung urologischer Palliativpatienten.
- Die SAPV ermöglicht den meisten Patienten im häuslichen Umfeld zu versterben und die häufigsten Symptome der Palliativsituation zu kontrollieren.
- Angehörige zeigen eine sehr hohe Zufriedenheit mit der SAPV.
- Eine gute Kooperation von urologischer (Palliativ)therapie und dem Angebot der SAPV könnte die Versorgung urologischer Tumorpatienten weiter verbessern.

## Data Availability

Alle relevanten Daten, die die Ergebnisse der vorliegenden Arbeit stützen, sind innerhalb des vorliegenden Artikels verfügbar. Weiterführende Daten sind beim erhebenden Verein VSTN e. V. auf plausible Anfrage beantragbar.
